# An Arrival and Departure Time Predictor for Scheduling Communication in Opportunistic IoT

**DOI:** 10.3390/s16111852

**Published:** 2016-11-04

**Authors:** Riccardo Pozza, Stylianos Georgoulas, Klaus Moessner, Michele Nati, Alexander Gluhak, Srdjan Krco

**Affiliations:** 1 Institute for Communication Systems, University of Surrey, Guildford GU2 7XH, UK; s.georgoulas@surrey.ac.uk (S.G.); k.moessner@surrey.ac.uk (K.M.); 2 Digital Catapult Centre, London NW1 2RA, UK; michele.nati@digicatapult.org.uk (M.N.); alex.gluhak@digicatapult.org.uk (A.G.); 3 Dunav Net, Novi Sad 21000, Serbia; srdjan.krco@dunavnet.eu

**Keywords:** internet of things, neighbour discovery, predictor, opportunistic, scheduling

## Abstract

In this article, an Arrival and Departure Time Predictor (ADTP) for scheduling communication in opportunistic Internet of Things (IoT) is presented. The proposed algorithm learns about temporal patterns of encounters between IoT devices and predicts future arrival and departure times, therefore future contact durations. By relying on such predictions, a neighbour discovery scheduler is proposed, capable of jointly optimizing discovery latency and power consumption in order to maximize communication time when contacts are expected with high probability and, at the same time, saving power when contacts are expected with low probability. A comprehensive performance evaluation with different sets of synthetic and real world traces shows that ADTP performs favourably with respect to previous state of the art. This prediction framework opens opportunities for transmission planners and schedulers optimizing not only neighbour discovery, but the entire communication process.

## 1. Introduction

The Internet of Things (IoT) [1] is an innovative paradigm gaining an increasing traction not only in the research community but also in the real world due to the pervasive diffusion of cheap and small IoT devices, estimated to generate an economic impact of up to $11.1 trillion per year by 2025 for IoT applications [2]. In such settings, digitally connected real world physical objects allow for a whole new lot of smart services and applications by relying on cross-applications sensors translating from physical quantities into “knowledge” [3] and on actuators capable of taking smart actions based on the learned experience.

Smart cities are just one of the envisioned IoT applications [4] because of the numerous problems that their councils need to face every day, impacting the lives of billions of people. Examples thereof are, just to name a few, water distribution, pollution control, public transportation and traffic management, street lighting and many more [5]. In such a scenario, the introduction of IoT devices and the definition of new services allows for an even higher number of possible smart applications [6] exploiting multiple sensing and actuation capabilities, as well as involving people in the process, e.g., fostering interaction between people and the government.

From a networking point of view, this scenario implies the need for increased connectivity between IoT devices and for a better way to manage their communications. While historically, in such settings, mobility of IoT devices has been seen as an added constraint into networking, more recently [7] it has been represented as an actual *opportunity* to convey information across different domains. In fact, it is well known that mobility can increase capacity [8] of traditional static networks not only by reducing congestion and improving reliability of delivery in multi-hop networks but also by promoting energy efficiency given the lower number of hops to transverse in order to reach destinations.

Opportunistic IoT [9,10] is therefore foreseen as a means to delivery data across disconnected islands of devices, where the presence of an end to end path between those never existed permanently. Mobile IoT devices are therefore considered as relaying devices which can store-carry-forward [11] across multiple networks. Evidently, in a Smart City, where a myriad of heterogeneous devices live and are deployed, bridging across multiple radio technologies opens up countless opportunities for new smart services and applications. For example, smartphones or more resource constrained wearables devices of people travelling on public transportation means (i.e., a bus) can encounter many devices such as sensors and actuators and forward data across Bluetooth, Wi-Fi and cellular networks via device-to-device (D2D) communications. In this scenario for opportunistic IoT, devices might not always be mobile but also static and battery-operated (i.e., roadside sensors/actuators) as well as lacking of readily available sources of power supply, thus requiring power management techniques in order to maximize their lifetime.

Resource constrained IoT devices with lower computational, storage or battery capabilities could assign heavier tasks to more powerful or easily rechargeable IoT devices [12] as well as allowing distributed processing amongst them. Evidently, in order to exploit such communication opportunities, IoT devices need to incorporate smart neighbour discovery protocols capable of optimizing at the same time the lifetime of IoT devices and not missing any meaningful contact [13]. Due to the availability of large datasets about people’s mobility, in the last years, it has been shown [14,15,16] that spatio-temporal patterns of urban mobility can be used to infer statistics about patterns of encounters between people. In particular, Song et al. [15] has shown that human mobility patterns present forms of regularity which allows for a potential 93% average predictability. Furthermore, due to the advent of Mobile Crowd Sensing and Computing paradigm [17], nowadays, it is indeed easier to exploit personal devices such as smartphones for running experiments such as crowdsourcing [18] anonymized data.

Recent research has shown that is possible to either learn [19,20,21] about such encounters or probabilistically, through statistical analysis, [22,23] adapt neighbour discovery in order to improve lifetime of devices and optimize communication time. We argue that the learning process is necessary in order to adapt online to the statistics of the pattern of encounters, and is optimizable by identifying the significant features of statistics of mobility such as spatial or temporal recurrence or contextual knowledge. While in the authors’ previous work CARD [21], neighbour discovery is adapted based on the acquired knowledge, in this work an algorithm for learning and predicting the actual values of “arrival” and “departure” of IoT devices is proposed.

In this paper, an algorithm for Arrival and Departure Times Prediction (ADTP) of IoT devices is presented, thus capable of also making estimates of durations of future encounters. The Least Squares Temporal Difference (LSTD) [24,25] learning algorithm is capable of making predictions relying only on temporal data, which require no energy expenditure to obtain, contrary to spatial data, typically obtained via GPS or accelerometer sampling, which need additional hardware that is not present in most resource constrained IoT devices. Moreover, the learning is performed *online* without requiring extensive training or data collection campaigns, and requires very few data and computational capabilities to output predictions. In addition, ADTP’s learning can take place both on the static and the mobile IoT devices, meaning that it is suitable for any type of resource constrained device, being it mobile or not. Finally, a short prediction errors history is used to recognize abrupt changes in mobility patterns and take immediate action to re-act to changes.

ADTP is evaluated against previous state-of-the-art extensively both in accuracy of prediction and in neighbour discovery performance on different synthetic and real world mobility traces under different mobility conditions typical of urban scenarios (i.e., controlled periodic, public transportation and human mobility based). The results show that ADTP outperforms previous state of the art in all scenarios taking into account both energy spent and discovery latency.

Especially concerning energy spent, the results show that ADTP introduces additional energy savings for both synthetic and real world traces and just increases its energy expenditure in order to keep a performance edge in latency over CARD in scenarios presenting a high degree of randomness, however still consuming very little energy if compared to other probabilistic state-of-the-art approaches. In fact, as reported in the evaluation section, if both wasted time and wasted energy for discovery are taken into account, a performance edge is shown for ADTP.

Most importantly, contrary to the previous work, ADTP can allow for optimization of the communication process since knowledge about contact times and durations can allow for adjusting the data transmission process accordingly, e.g., not starting a data transmission if the contact time will mean it will fail midway. This allows for additional saving energy and bandwidth due to the avoided failed transmissions and retransmissions.

The remainder of this publication is organized as follows. Section 2 reviews the scenario and current state-of-the art for discovery in opportunistic IoT. Section 3 introduces the arrival and departure prediction and communication scheduling model. Section 4 reports the results obtained through an extensive set of simulations for performance evaluation. Section 5 concludes the publication and discusses about future work.

## 2. Background and Related Work

### 2.1. Scenario of Opportunistic IoT

**Figure 1:**
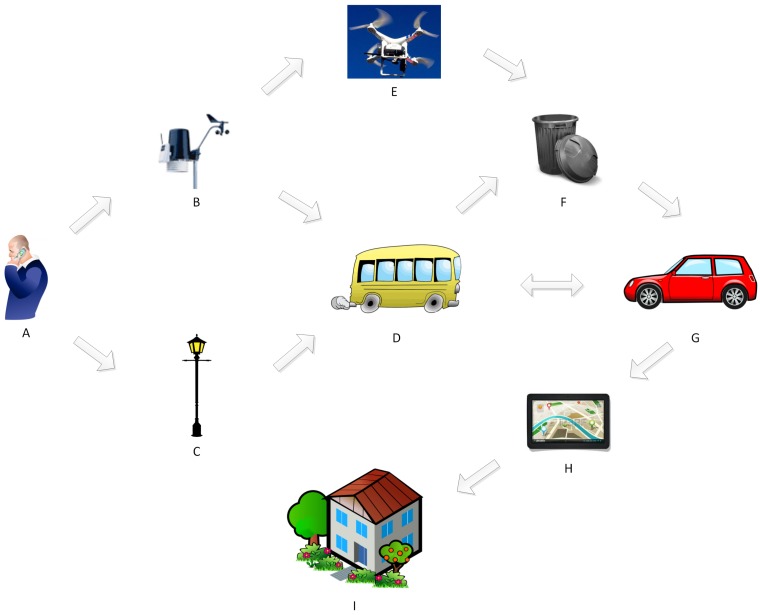
Opportunistic Internet of Things (IoT) in a Smart City.

Urban scenarios typical of a Smart City enable a new series of proximity services due to the opportunistic nature of interactions between mobile and static IoT devices such as smartphones, wearables and wireless sensors/actuators. A typical scenario such as the one depicted in Figure 1 includes people willing to share their devices and undertake the additional power consumption burden needed for opportunistic communication (A), autonomous vehicles following a controlled mobility (E) and public transportation based devices (D) all acting as proxies for data collection, dissemination, storage and forwarding. In addition, wireless sensors deployed along the roads (B) or inside garbage bins (F) as well as wireless actuators such as street lamps (C) have opportunities to interact with the aforementioned mobile IoT devices, thus avoiding the cost of deployment and maintenance of networking infrastructure for their internet connectivity.

Even though some IoT devices (i.e., cars, buses, street lamps) have readily available power supplies, other devices such as i.e., mobile data collectors attached to bicycles or livestock, static sensors/actuators deployed in garbage bins, drones and smartphones might rely on efficient power management mechanisms for prolonging their lifetime and not impacting user experience. It is important to note also that, given the heterogeneous nature of IoT devices, many devices might incorporate multiple radios, thus acting as bridges for opportunistic communication, but also needing to manage additional radios and the associated multiple power consumption to be able to discover different networks. The diversity and heterogeneity of IoT radio technologies further impacts the number of transmission opportunities in dense deployments such as Smart Cities, where many devices might not implement Wi-Fi but other low power radio technologies, thus requiring to rely on other IoT devices as relays.

Opportunistic IoT in urban scenarios allows for new applications affecting globally citizens around the world. For example, a person (A) could collect data about pollution/noise [26] (B) and forward such data to other people in buses (D) or be collected by drones (E) and ultimately relayed to other people in cars or taxi cabs [27] (G) to enable a navigation application (H) to optimize the route to home (I) based on traffic/congestion information. Such data could be combined with data about fullness of recycling bins (F) relayed by people (G, D) to enable smart collection routes for garbage trucks. Finally, an S.O.S. health application deployed in a smartphones (A) could trigger street lamps (C) to signal attention to nearby people or simply to save power by dimming the lamps off when no one is walking nearby.

### 2.2. Neighbour Discovery

In this scenario of Opportunistic IoT, mobility plays the important role of enabler of pervasive communication. By correctly recognizing opportunities for communication, neighbour discovery techniques based on knowledge about patterns of mobility [13] are in fact able to exploit the nature of opportunistic IoT scenarios in their favour to optimize the scheduling of their communication. While approaches exploiting spatial knowledge about mobility acquired from accelerometers and geographical location sampling potentially extract more information about the mobility context, in this work the focus is only on approaches exploiting temporal features of mobility as deemed both more generally applicable and not involving power consumption in order to gather such knowledge. In fact, spatial knowledge based approaches require additional hardware just to extract such information.

Temporal mobility knowledge can be obtained just by gathering statistics about temporal features between IoT devices, computed in a distributed manner on each device. For example, the adaptive energy conserving algorithms by Drula et al. [28] for opportunistic Bluetooth networks dynamically change the protocol parameters defining the discovery times according to the level of activity seen by the device. Similarly, the adaptive exponential beaconing (AEB) protocol by Choi and Shen [29] exponentially relaxes the discovery times as the trend of contact availability decreases over time. The Short Term Arrival Rate (STAR) estimation by Wang et al. [22] proposes instead to adapt the contact probing interval dynamically based on the arrival rate, statistically estimated with minimal error, accordingly to the previous time-of-day or time slot information.

Han and Srinivasan [30] introduce eDiscovery, an adaptive inquiry algorithm which modifies the Bluetooth protocol parameters based on the increase or decrease of the number of peers discovered. Zhou et al. [31] describe an adaptive working schedule based on a time slotted model which infers the expected encounter levels to be seen in future slots, therefore adapting according to the rate of next arrivals. Finally, Wi-Fi Sensing with AGing (WiSAG) by Jeong et al. [32] proposes to adapt the sensing times according to the characteristics of the inter contact times and contact durations, namely an aging property that, if negative should allow more sleeping, and otherwise, if positive.

Chakrabarti et al. [33] report of using predictable device mobility in sensor networks in order to improve power efficiency in communications. In such a work, the knowledge about the mobility pattern of the mobile device is acquired in a startup phase and then exploited to save energy. Jun et al. [34] introduce a framework for power management based on different levels of knowledge about mobility patterns, showing that significant energy savings can be achieved by knowing statistics (mean and variance) about inter contact times and contact durations. In a later work [35], the authors propose a hierarchical radio approach which combines low range but low power radios with high range but high power radios optimized according to traffic load knowledge.

Dyo and Mascolo [19] instead adopt a reinforcement learning based approach capable of learning the expected encounter frequency by getting reinforcements from across subsequent days in time-of-day slots and by adapting the probing frequency accordingly. A framework for resource aware data accumulation (RADA) is presented by Shah et al. [20], also exploiting reinforcement learning by making the static device learn to schedule higher duty cycles (i.e., proportion of time the radios are on in periods) according to inter contact times and time-of-day information, thus increasing chances for discovery.

Sensor Node Initiated Probing for Rush Hours (SNIP-RH) by Wu et al. [36] concentrate more effort during rush hours, when a higher probability to find neighbouring devices is foreseen based on average contact duration. Kondepu et al. [37] instead propose an approach where transmission power is modified to send short and long range beacons. Their work learns to schedule a higher duty cycle of short range beacons and a lower duty cycle of long range beacons, based on the approaching mobile element range as seen by the static device.

The work by Gao and Li [23] defines a Probabilistic Wakeup Scheduler (PRWS) based on stochastic modelling of the node contact process, which improves over the adaptive contact probing of STAR [22]. The framework allows for wakeup scheduling based on a strategy which allows for sleeping in between contacts and only wake up nodes when they are predicted to be in contact with high probability. Similarly, Zhang et al. [38] exploit the power law statistical property of inter contact times to define a wakeup scheduling which allows for matching contacts with high probability.

Finally, the Context Aware Resource Discovery of Pozza et al. [21] reports an algorithm which learns the optimal schedule in order to jointly optimize energy efficiency and discovery latency. This discovery approach exploits Q-Learning [39] to learn by trial-and-error the optimal sequence of discovery action, composed by low latency sub-actions and high latency sub-actions, which maximizes the long term reward driven by contact discoveries. The approach indeed tries to match low latency sub-actions when the contact is learned to be expected while scheduling high latency sub actions when it is not.

## 3. Prediction and Scheduling Model

**Figure 2:**
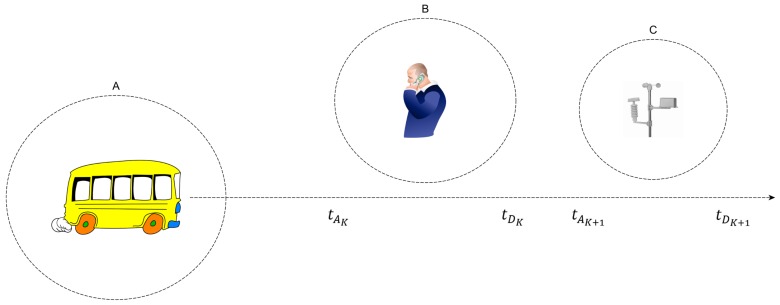
Temporal Prediction of contacts between mobile IoT devices (**A**,**B**) and static IoT devices (**C**).

ADTP allows to make predictions about future encounters of an IoT device based on the learned temporal pattern of interactions with other devices. An example prediction scenario is presented in Figure 2. It is important to note that ADTP allows to make predictions not only of the immediately next contact, but also future contacts though, with a lower degree of accuracy. For example, the bus (A) and the smartphone (B) both predict the “be within radio communication range” (represented by the dashed line) between $t_{A_{K}}$ and $t_{D_{K}}$. In addition, the bus is also able to infer the subsequent contact with the sensor (C) to occur between $t_{A_{K+1}}$ and $t_{D_{K+1}}$.

The algorithm therefore provides estimates about when in time and for how long a contact will occur with high probability in the future, based on the knowledge acquired over time about the encounters already occurred. This is achieved by learning about contact arrival and departure times, which also allows to compute the forecasted contact duration as their difference. Based on such predictions, ADTP plans for every contact an optimal discovery action scheduling which allows for additional savings with respect to previous state-of-the-art. In that work, a trial-and-error action scheduling, composed also of sub-optimal actions, leads to learning the optimal sequence of actions which maximizes the reward, proportional to the latency with which a contact is discovered. It is to note that no spatial knowledge is used since it would require additional hardware (and power consumption) to gather such knowledge, thus impacting the applicability range of ADTP.

ADTP’s predictions are used in this work to perform a low latency fast discovery when a contact is foreseen with high probability, (e.g., close in time to $t_{A_{K}}$) combined with a power saving high latency schedules when contacts are forecast with very low probability (e.g., between $t_{D_{K}}$ and $t_{A_{K+1}}$). Moreover, selective sleeping is enforced when predictions are accurate over time, thus allowing for additional power savings. At the basis of ADTP there is a general framework, applicable to any kind of IoT device, being those resource constrained or more powerful such as smartphones. In fact, by being based on reinforcement learning methods which require low computational capabilities, no training phases and few data, it can be used even in low power IoT devices. Moreover, ADTP uses a deterministic temporal overlap protocol (see taxonomy in [13]) as its underlying neighbour discovery protocol, thus providing latency guarantees and no need for time synchronization. Finally, since in some situations mobility pattern could change abruptly, an adjustment of the learning process is performed based on a measure of the accuracy of predictions.

### 3.1. Temporal Difference Learning

Common applications of reinforcement learning include robotics, games, human-computer interactions and financial economics. In fact, the main objective in such settings is to learn how to control the behaviour of some agent in a real world environment, guided by positive or negative rewards for performing actions over time. However, as Sutton proposed in his pioneering work [40], temporal difference methods where initially conceived as a means of prediction about a value which is reinforced over time. Examples thereof are weather prediction and financial market forecast, where a value is predicted and sampled and refined over time as more data becomes available.

After an investigation, it was actually observed that the temporal evolution of mobility an IoT device follow is not influenced by the choice of the IoT device’s actions but it is actually the owner or user of such IoT devices that influences its mobility, i.e., someone’s walking route to work, the public transportation means route. This implies that the learning environment fits better that of a *policy evaluation* environment in which the agent’s actions are not influenced by the trajectory in the state space, being there no *policy improvement* steps.

Conceptually, the objective of the temporal difference prediction framework is to learn in a step-by-step process a value function named $V^{\pi }$ result of evaluating a particular policy *π*. For example for the state $s_{t}$ at step *t*, the value function is updated as such:

(1) $$V\left(s_{t}\right)\leftarrow V\left(s_{t}\right)+\alpha \left[R_{t}-V\left(s_{t}\right)\right]$$

where *α* represents the step-size parameter or *learning rate* (a measure of how fast new information is incorporated) and $R_{t}$ is the reward observed at step *t*. In the n-step case, such a reward is equal to a discounted sum of future rewards:

(2) $$R_{t}^{\left(n\right)}=r_{t+1}+\gamma r_{t+2}+\gamma^{2}r_{t+3}+\ldots +\gamma^{n-1}r_{t+n}+\gamma^{n}V_{t}\left(s_{t+n}\right)$$

where $r_{t+n}$ is the reward observed at the t+n-th step, $V_{t}\left(s_{t+n}\right)$ is the estimate of the value function in state $s_{t+n}$ and *γ* is the *discount factor* (a weight on how much future rewards influence the current value function).

The temporal difference learning methods make also use of *eligibility traces*, which is a mechanism for allowing averaged long term rewards to backpropagate based on the 0≤λ≤1 parameter as such:

(3) $$R_{t}^{\left(\lambda \right)}=\left(1-\lambda \right)\sum_{n=1}^{\infty }\lambda^{n-1}R_{t}^{\left(n\right)}$$

The parameter *λ* indeed sets the decay speed of the rewards propagation, with the degenerate case of λ=0 corresponding to a 1-step update where only the immediate reward influences the value function. Conversely a value of λ=1 means the updates are influenced by all the evolutions in the state space, thus similarly to a Monte Carlo approach.

Since ADTP’s framework aims at predicting temporal values, which might cause the state space to be large, *function approximation* for the value function was introduced, resulting in:

(4) $$V^{\pi }\left(s\right)\approx \theta \;\cdot \;\phi \left(s\right)$$

where ϕ(s) is a *feature representation* in the state space and *θ* is the *parameters vector* to be learned. The parameters vector is updated at every iteration as such:

(5) $$\theta \leftarrow \theta +\alpha_{n}\delta$$

where $\alpha_{n}$ represents the learning rate for the n-th *episode* considered (a trajectory in the state space until a terminal state) and *δ* represents the *temporal difference* update. This is computed as such:

(6) $$\delta \leftarrow \delta +\Delta \theta_{t}$$

where $\Delta \theta_{t}$ represents the temporal difference error. The temporal difference error is function of the difference between the value function in subsequent states, of the reward and of the eligibility traces, thus leading to the update:

(7) $$\Delta \theta_{t}=e_{t}\left[R_{t}+{\left(\gamma \phi \left(s_{t+1}\right)-\phi \left(s_{t}\right)\right)}^{T}\theta \right]$$

where the eligibility traces weight the different feature vectors as such:

(8) $$e_{t}=\sum_{k=1}^{t}\lambda^{t-k}\phi \left(s_{k}\right)$$

The Least Squares Temporal Difference (LSTD(*λ*)) algorithm for approximate policy evaluation (see Boyan [41]) capable of learning the parameters vector *θ*, is represented in Algorithm 1. Different from TD(*λ*), which performs stochastic gradient descent on a cost function of the parameters, LSTD(*λ*) builds estimates of (a constant multiple of) a vector *d* and a matrix *C*, solving a system of equations as such:

(9) $$d+C\theta_{\lambda }=0$$

**Algorithm 1:** LSTD(*λ*) for approximate policy evaluation - Boyan (1999)
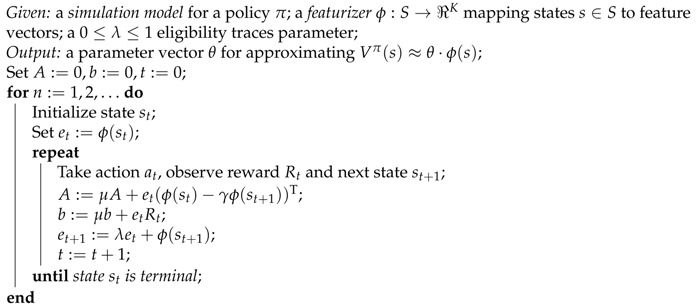


The algorithm constructs a vector *b* and a matrix *A* as unbiased estimates over *n* episodes of, respectively nd and −nC, as such:

(10) $$b=\sum_{i=0}^{t}e_{i}R_{i}$$

and:

(11) $$A=\sum_{i=0}^{t}e_{i}{\left(\phi \left(s_{i}\right)-\phi \left(s_{i+1}\right)\right)}^{T}$$

As it can be seen from Algorithm 1, at every step the matrix *A* and the vector *b* are updated recursively. The parameters vector can be obtained by a simple matrix inversion (by Singular Value Decomposition) and a matrix-vector product as follows:

(12) $$\theta :=A^{-1}b$$

Finally, the parameter 0≤μ≤1 represents an *exponential windowing factor* [42], which works as an exponential decay for updates, thus meaning that depending on the value, far or less distant updates have more or less weight.

### 3.2. Arrival and Departure Time Predictor

**Figure 3:**
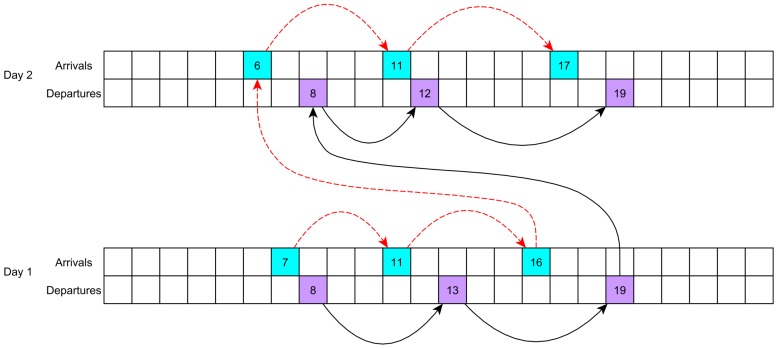
Example of arrival and departure times evolution in the state space.

Figure 3 shows an example about the trajectory in state space for the the arrival and departure time predictors, which helps in understanding the modelling adopted for the predictor. For example, an evolution in the state space for the arrivals goes from state 7, corresponding to 7 AM in the morning to state 11, to state 16, and then back to state 6, state 11 and state 17. In a similar way, the evolution of departure times in the state spaces proceeds independently in parallel, from state 8 to 13, 19, 8 again, 12 and 19. Every action of users of mobile devices in the real world will thus lead to states defined as:

(13) $$s_{A_{k}}\in S_{A};\;\;\;\;\;\;\;\;\;\;\;\;\;\;\;s_{D_{k}}\in S_{D}$$

As aforementioned, the value functions are approximated by two vectors:

(14) $$V_{A}^{\pi }\approx \theta_{A}\;\cdot \;\phi_{A};\;\;\;\;\;\;\;\;\;\;\;\;\;\;\;V_{D}^{\pi }\approx \theta_{D}\;\cdot \;\phi_{D}$$

(15) $$\theta_{A}=\left[\theta_{A_{0}},\theta_{A_{1}}\right];\phi_{A}=\left[1,\phi_{S_{A}}\right];\;\;\;\;\;\;\;\;\;\;\;\;\;\;\;\theta_{D}=\left[\theta_{D_{0}},\theta_{D_{1}}\right];\phi_{D}=\left[1,\phi_{S_{D}}\right]$$

where $\phi_{S_{A}}$ and $\phi_{S_{D}}$ represent the arrival and departure times. Since in this framework it is of interest only to predict the next arrival and departures, the value function is used to learn next arrivals and departures times. It is important to note though that this choice does not affect the capability of the algorithm to predict multiple contacts ahead, just by evaluating the trajectory in the predicted state space through the value function.

In a next contact prediction environment, the current prediction does not intuitively depend on the next prediction, so the discount factor was set to γ=0. Similarly, future rewards are not backpropagated in order to influence the learning, thus leading to a eligibility trace parameter λ=0. The rewards are then defined accordingly to the observed arrival and departure times:

(16) $$r_{A_{t}}=\phi_{S_{A_{t}}};\;\;\;\;\;\;\;\;\;\;\;\;\;\;\;r_{D_{t}}=\phi_{S_{D_{t}}}$$

In order to recognize sudden changes in mobility patterns such as a change of pattern between weekdays and weekends, the arrival predictor is equipped with a history of the errors between the predicted values and the values actually observed. A simple moving average of the errors history is used to evaluate the accuracy of the predictions, thus identifying diverging trends. In particular, at step *t*, considering a history of $N_{E}$ samples, for the moving average it follows:

(17) $$E_{t}=\frac{1}{N_{E}}\sum_{k=0}^{N_{E}-1}\left(\phi_{S_{A_{t-k}}}-P_{S_{A_{t-k}}}\right)$$

where $P_{S_{A_{t}}}$ is the prediction at step *t* obtained by evaluating the value function. At every step, a comparison between the moving average $E_{t}$ and $E_{t-\frac{N_{E}}{2}}$ is made and, if 50% greater, an abrupt change of mobility pattern is considered in place. This triggers a change in the exponential windowing factor, which is temporarily lowered to $\mu_{min}=0.3$ and raised to $\mu_{max}=0.9$ in Δμ=0.1 increases per step. As a consequence, a behaviour which tries to weight less previous remote updates than closer updates, thus acquiring fresher knowledge is obtained.

### 3.3. Resource Discovery Planner and Scheduler

**Figure 4:**
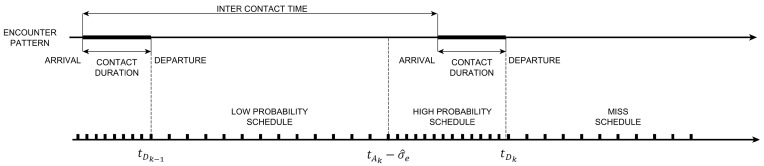
Resource Scheduler based on Arrival and Departure Predictions.

ADTP’s resource scheduler is based on the arrival and departure times predicted by the two instances of LSTD(*λ*) running in parallel and receiving new rewards for every discovered IoT device. Figure 4 illustrates the encounter process and all the relevant metrics involved in the temporal evolution of such a contact pattern as well as how the resources are scheduled from the point of view of a device in order to optimize the discovery process. In particular, it is possible to identify three different possible schedules:

- *Low Probability Schedule* (LPS) to be scheduled when contacts are predicted with lower probability thus introducing energy savings.
- *High Probability Schedule* (HPS) to be scheduled when contacts are predicted with high probability thus allowing for a timely discovery.
- *Miss Schedule* (MS) to be scheduled when discovery did not happen in previous schedules.

In order to define the boundaries of such schedules, along with the next predicted arrival time $t_{A_{k}}$ and next predicted departure time $t_{D_{k}}$, a mean square prediction error estimate $\hat{\sigma_{e_{k}}}$ is computed as such:

(18) $$\hat{\sigma_{e_{k}}}=\sqrt{\frac{1}{N_{E}}\sum_{i=0}^{N_{E}-1}{\left(\phi_{S_{A_{k-i}}}-P_{S_{A_{k-i}}}\right)}^{2}}$$

This measure allows to quantify the uncertainty about the predictions in order to match the contact arrival with a HPS. As it can be seen from Figure 4, the LPS is scheduled since the last departure $t_{D_{k-1}}$ until the next predicted arrival $t_{A_{k}}$ minus the deviation of error estimate $\hat{\sigma_{e_{k}}}$. Afterwards, a HPS is scheduled until the next predicted departure $t_{D_{k}}$ and, if a contact is found, a new LPS and HPS iteration is made. However, if a contact is not discovered, the MS is scheduled until a new discovery is made, thus triggering a new LPS and HPS iteration.

Similarly to CARD [21], the actions for LPS and HPS are, respectively high latency and low latency actions as such:

- *High Latency Action* (HLA) guaranteeing discovery within a temporal bound $t_{HLA}=D$.
- *Low Latency Action* (LLA) guaranteeing discovery within a temporal bound $t_{LLA}=0.05\;\cdot \;D$.

where the parameter *D* is decided by application requirements. As in CARD, the general temporal overlap driven approach by Dutta et al. [43] was used, relying on prime numbers properties to guarantee overlap between asynchronous nodes. In particular, given the latency bounds of above and the slot time $t_{\mathrm{slot}}$, the scheduler computes the *candidate* prime value as:

(19) $$p=\sqrt{\frac{t_{\mathrm{bound}}}{t_{\mathrm{slot}}}}$$

and then builds a *Sieve of Atkin* in order to obtain a balanced prime pair $p_{i},p_{j}\leq p$ such that:

(20) $$t_{{\mathrm{bound}}^{\prime }}=p_{i}\;\cdot \;p_{j}\;\cdot \;t_{\mathrm{slot}}\leq t_{\mathrm{bound}}$$

An additional feature named *selective sleeping* was introduced in order to introduce additional energy savings by allowing to selectively sleep during the next LPS schedule according to the correct happening of a discovery during the last HPS schedule. This rewards the resource scheduler with lower power consumption when the predictions are accurate.

Finally, it was noticed that when accuracy is very high a *drift* effect is in place, caused by a restriction of the temporal window for the HPS schedule. As a countermeasure, it was introduced a lower bound on $\hat{\sigma_{e_{k}}}$ as follows:

(21) $$\hat{\sigma_{e_{\min}}}\geq p_{i_{\mathrm{LLA}}}\;\cdot \;p_{j_{\mathrm{LLA}}}\;\cdot \;t_{\mathrm{slot}}$$

equivalent to the minimal duration for a guaranteed discovery with low latency. Moreover, when the contact is very short, the predictor might output a $P_{S_{A_{t}}}\geq P_{S_{D_{t}}}$, impacting our implementation. In such cases, ADTP computes new values as follows:

(22) $$P_{S_{A_{t},D_{t}}}=\frac{P_{S_{A_{t}}}+P_{S_{D_{t}}}}{2}\pm \frac{\hat{\sigma_{e_{\min}}}}{2}$$

Thus as an average and spanning the minimal duration discussed above.

## 4. Results

This section introduces the results obtained by evaluating ADTP’s performance, both in the accuracy of the predictor and in the energy and latency trade-off of its resource discovery planner scheme.

### 4.1. Predictor Evaluation

**Figure 5:**
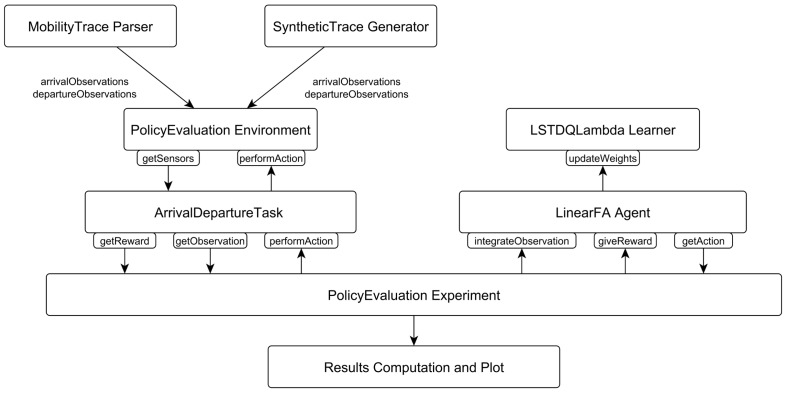
ADTP implementation on PyBrain.

The design of ADTP and an evaluation of its accuracy has been carried out by relying on the Python-based Reinforcement Learning, Artificial Intelligence and Neural Network (*PyBrain* [44], Dalle Molle Institute for Artificial Intelligence, IDSIA, Switzerland and Technische Universitat Munchen, Germany) library. In Figure 5 it is possible to see the implementation adopted for the framework, which evaluates different synthetic and real world mobility traces.

The *SyntheticTrace Generator* creates arrival and departure times for *Deterministic*, *Multiple Deterministic*, *Gaussian* and *Multiple Gaussian* traces, previously used for CARD [21] and corresponding to controlled/robotised mobility (i.e., Unmanned Aerial Vehicles (UAVs), autonomous data collectors vehicles) and public transportation means (i.e., buses, trains). The Deterministic traces represent a fixed inter contact time of 30 min, while the Multiple Deterministic traces just add an increase of 3 min every 2 days. Similarly, the Gaussian and Multiple Gaussian normally distribute the previous inter contact times according to a ±3σ of 15 min.

The *MobilityTrace Parser* instead generates the arrival and departure times according to the mobility patterns of traces collected during an in-house experiment [45] representing Bluetooth sightings and passive infrared (P.I.R.) sensor based presence detection in an office environment, as well as the traces collected during the Haggle Project [46] representing interactions in a conference (Infocom) and laboratory environment (Cambridge Computer Lab and Intel Research Lab).

The *PolicyEvaluation Environment* is in charge of feeding the observations after every *performAction* and subsequent *getSensors* call executed by the *ArrivalDeparture Task*. The PolicyEvaluation Experiment is then in charge of retrieving a new action through *getAction* at every step and calling the *performAction* method of the ArrivalDeparture task, thus triggering the computation of a new reward via *getReward* and a new observation via *getObservation*. The observation and reward are then integrated via *integrateObservation* and *giveReward* methods to the *LinearFA Agent* which then updates the weights on the *LSTDQLambda Learner* via the *updateWeights* method. At the end of the experiments, the metrics of interest are parsed and plotted.

In Table 1, it is possible to see the results of the accuracy of the predictor of the arrivals and the departures. These are the percentage of discovered contacts over the total number of contact experienced during the simulation time, which is of 20 days for the synthetic traces, about a month time for the in-house traces and a week for the Haggle traces.

At a glance, it is possible to see coherence between the results for the arrivals and the departures across all traces. For the Deterministic traces, the predictor converges to the actual observations in few steps, thus leading to 99.8% of the predictions within 1 min. In the Multiple Deterministic case, the change in periodicity introduces a few more learning steps with less accuracy, but leads to a still very high accuracy of 96.4% predictions within 1 min of the observations. For the Gaussian and Multiple Gaussian traces, it is possible to see a percentage of predictions distributed coherently with the normality of the distribution, meaning the predictor is still able to predict the arrivals, though mainly on average.

Concerning the real world traces, it was found that Bluetooth traces are the best in terms of accuracy with 82.5% of predictions within 5 min. The P.I.R., Intel, Cambridge and Infocom traces, instead average around 50% of predictions within 10 min. This observed dichotomy is most likely due both to the fine-grained resolution of the Bluetooth traces and on the longer period of collection, which results in an higher number of short contacts.

In Table 2, it is possible to see the predictions of the two steps ahead arrivals and departures. It is possible to note an expected symmetric degradation of performance, quantifiable in a roughly 10% less accuracy in the predictions on average. Even though the accuracy is degraded, this scenario opens up the path to planning communication in advance, due to the capability to predict indirectly as a difference not only contact duration, but also multiple steps ahead. For example, as aforementioned, TCP tuning, radio interface selection based on speed and duration of contacts and caching for opportunistic content dissemination are only a few of the applications that can be envisioned.

**Figure 6:**
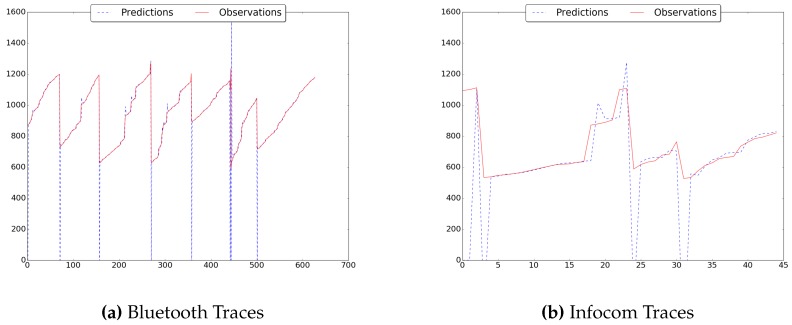
Predictions and Actual Observations.

Finally, in Figure 6 it is possible to see how the predictor matches the observations of the arrival times for two real world traces. In particular, it is possible to see that for the Infocom traces between the 15-th contact and the 35-th contact four abrupt variations challenge the predictor, thus leading to a lower accuracy with respect to, e.g., the Bluetooth traces.

### 4.2. Planner and Scheduler Evaluation

**Figure 7:**
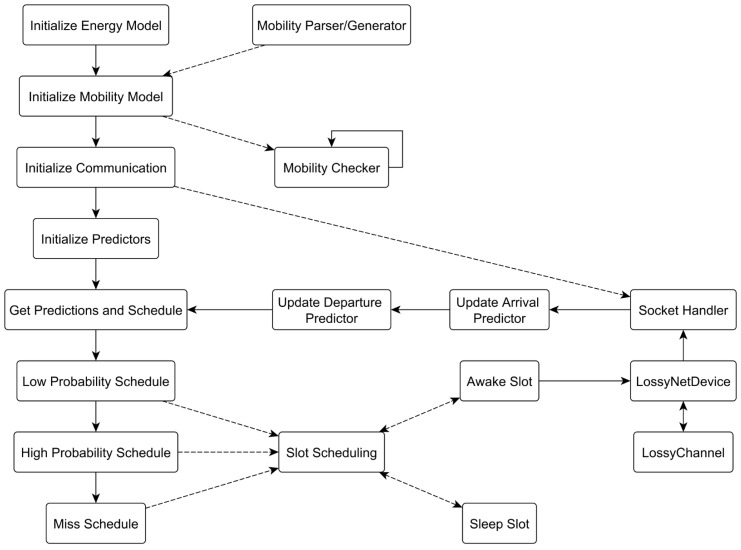
ADTP implementation in NS3.

Figure 7, shows the implementation of ADTP under the network simulator NS3 (NS-3 Consortium: French Institute for Research in Computer Science and Automation, Rocquencourt, France and University of Washington, WA, USA) [47]. At the beginning the application starts by initializing the energy model with the parameters of a CC2420 radio (Texas Instruments, Dallas, TX, USA) [48]. These are the three states of 19.7 mA of trasmission current, 17.4 mA of reception current and 1 μA of standby current, as well as 3 V of supply voltage.

The application then initializes the mobility model according to the traces generated by the mobility parser (for the real world traces) or the generator for the synthetic traces. The mobility traces used consist of the Deterministic and Multiple Deterministic traces with a fixed inter contact time of 30 min, plus the eventual 3 min increase every two days. In addition, Gaussian and Multiple Gaussian traces were simulated, distributing inter contact times according to a ±3σ of 2.5 min. These traces were simulated in three different speeds of 3.6 km/h (human walk), 20 km/h (slow vehicle) and 40 km/h (fast vehicle) representing three different contact durations of 200 s, 36 s, 18 s with a radio range of 100 m. The real world traces, instead are the same as in the previous section. A mobility checker is then instantiated to check the range of interactions between devices and log performance metrics concerning energy, latency and discovery ratio. Then, the communication is initialized, by registering callbacks for socket based communication. A lossy channel is used as the communication means, which models the selected (see [49]) propagation loss as the Log Distance model and the fading loss as the Nakagami-m Fast Fading model. The LossyChannel also features a −90 dB energy detection threshold and a 0 dBm transmission level.

The arrival and departure predictors are then instantiated and initialized with the starting values. It is important to note that the predictor is build based on the Armadillo C++ library (Australia’s Information and Communications Technology Research Centre of Excellence, Sydney, Australia) [50] in order to help with linear algebra computation, but this is not necessary in principle on resource constrained devices as the only complex operation is a 2-by-2 matrix inversion. The predictor is then queried to retrieve the actual values and setup the communication schedule for the next contact, in particular, concerning the primes to be used for the slotted asynchronous discovery. Firstly a LPS is scheduled, then the HPS and eventually a MS, in case no discovery has took place during the previous two schedules. Within each of them, primes are used to count 10 ms length slots (with beacons of 1 ms) and eventually schedule packet communication if awake slots or sleep otherwise.

Additionally, the probabilistic wakeup scheduler (PRWS) of Gao and Li [23] was selected as a state-of-the-art comparison reference for probabilistic methods, since it improves over previous work STAR by Wang et al. [22]. The approach was implemented and compared, by letting it schedule either a HPS when supposed to be awake or a sleeping period when in between contacts. In particular, a probabilistic predictor was developed, capable of generating the appropriate predicted schedule by exploiting at every step iterative numerical methods leveraging C++ boost libraries mathematical tools [51].

A simulation set of 50 independent parallel runs [52] with 95% confidence intervals has been performed, evaluating the metrics as the average latency for discovery in percentage with respect to the contact duration and the energy spent for discovery while out of contact, as well as a metric combining the product of wasted time (the sum of latencies) and energy for discovery. This last metric gives an indication of how well a scheme works overall considering that, in order to perform well, it should jointly reduce latency and, at the same time, minimize energy spent. For example an always on scheme would achieve the lowest discovery latency but with the worst energy spent, which would reduce the merit of discovering with the lowest latency. The simulation ran for an equivalent time corresponding to 10 days for the synthetic traces, whereas, for the real world traces, corresponding to their actual duration.

**Figure 8:**
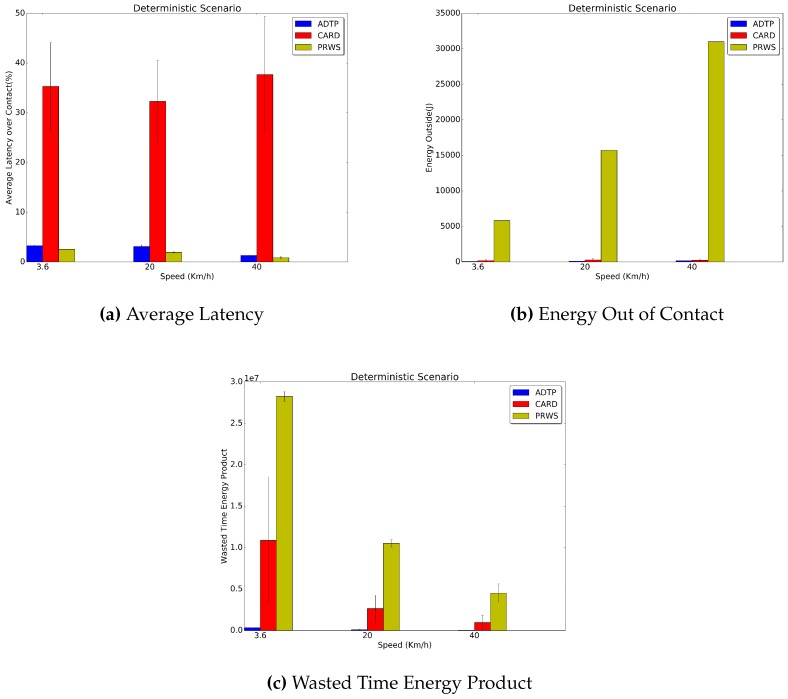
Deterministic Traces Results.

In Figure 8 it is possible to see the average discovery latency and the energy consumption while out of contact for the Deterministic traces. ADTP has a discovery latency which on average across all speeds is 32.5% lower than the latency of CARD. As for the energy consumed for probing out of contact, it is possible to see that for longer contacts ADTP consumes as much as 71.4% less energy than CARD, while needing 28.2% less energy for shorter contacts at 40 km/h. Concerning the evaluation against PRWS, it is possible to see that it achieves the lowest latency, but the energy spent by this approach in order to discover all contacts is far greater than the one needed by both ADTP and CARD. For example, at 3.6 km/h ADTP consumes roughly 99.1% less energy than PRWS, with CARD consuming as well 96.8% less energy than PRWS. The performance edge of ADTP and CARD is also clearly reflected in the wasted time energy product, which, in the worst case of 3.6 km/h leads to a 98.4% lower value for ADTP and a 46.3% lower value for CARD with respect to PRWS.

**Figure 9:**
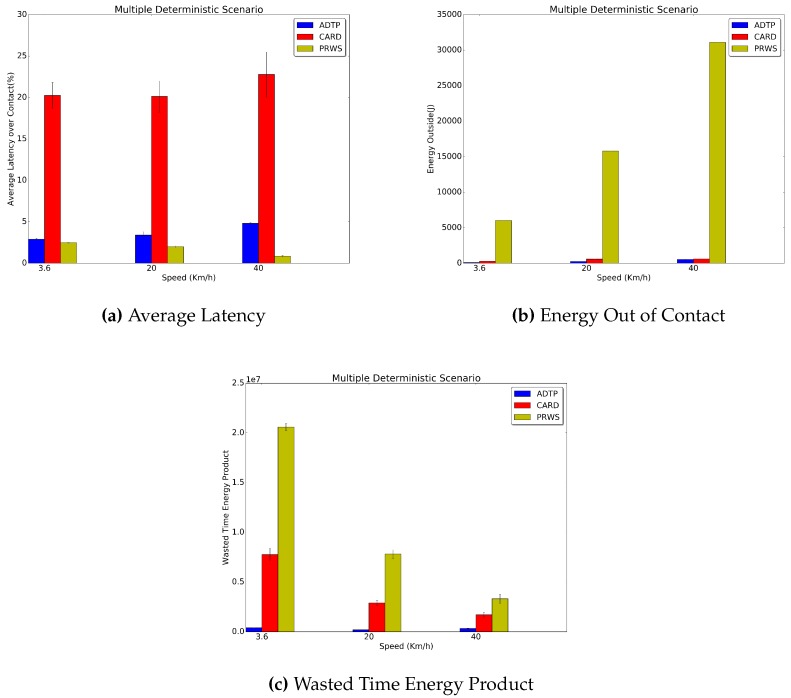
Multiple Deterministic Traces Results.

Figure 9 reports the results obtained for the Multiple Deterministic traces. It is possible to see that the periodic perturbation of periodicity in the traces reduces the performance of ADTP, on average, to a 17.3% less than CARD’s average latency. Similarly, for the energy consumed while out of contact, the improvement of ADTP over CARD is as high as 64.3% lower energy for the 3.6 km/h case to as low as 11.05% lower energy for the 40 km/h case.

This is due to the fact that the 3 min perturbation of periodicity is ten times bigger than the best possible contact duration (18 s), thus leading to a more challenged prediction. Indeed, these changes in mobility patterns, lead to a longer HPS schedule and therefore to higher energy expenditure.

As regards PRWS, it is possible to see that the energy spent by this approach in order to discover all contacts with the lowest latency is still far greater than the one needed by both ADTP and CARD. In particular, in the 40 km/h worst case ADTP consumes an energy which is 98.3% less than the energy of PRWS, with CARD consuming as low as 95.4% less than PRWS in the 3.6 km/h case. Concerning the wasted time energy product it is still possible to see a performance edge of ADTP and CARD with respect to PRWS, quantifiable in a 90.6% lower value for ADTP and in a 48.5% lower value for CARD in the worst 40 km/h case.

**Figure 10:**
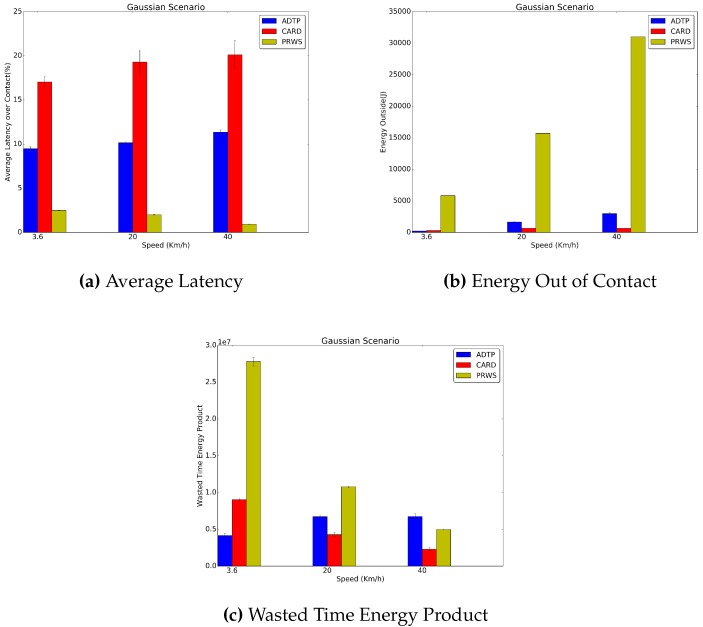
Gaussian Traces Results.

**Figure 11:**
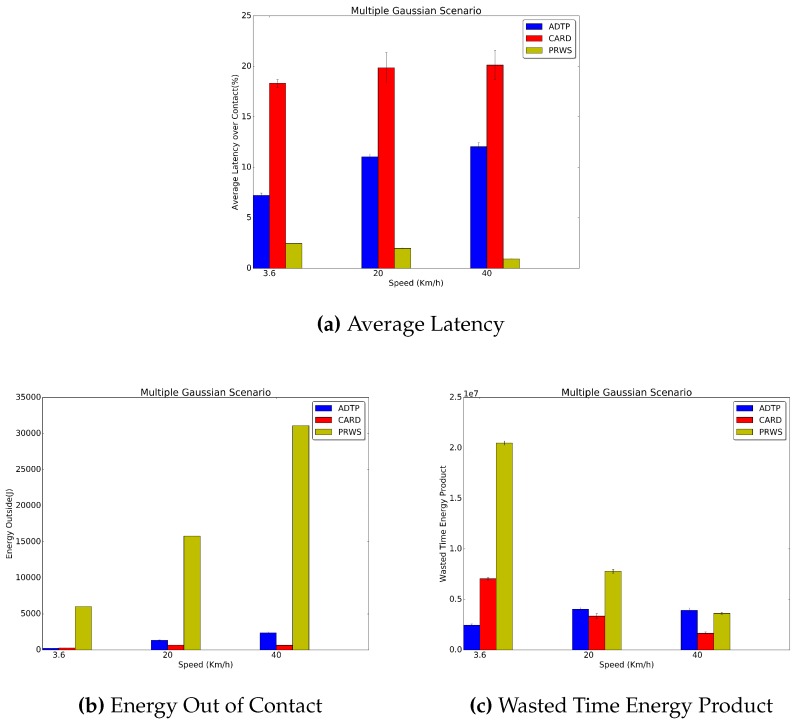
Multiple Gaussian Traces Results.

In Figure 10 and Figure 11, it is possible to see the results for the Gaussian and Multiple Gaussian traces. Concerning the average latency, across all contact durations, ADTP has an advantage of on average 8.5% for the Gaussian traces and of 9.3% for the Multiple Gaussian traces.

Additionally, by considering that in the gaussian scenario all the contacts are normally distributed within 300 s at 99.7% of the fixed intercontact time and that the contact durations are 200 s, 36 s and 18 s, it is possible to understand why there is a sharp increase in power consumption as the contact duration decreases.

In fact the Gaussian traces randomness pose a great challenge on the predictor capability to predict contact displacements, with the predictor only able to predict those on average and increasing the length of the HPS schedule, therefore dynamically increasing power consumption to keep a lower latency supremacy. In fact, only in the 3.6 km/h case, ADTP has a lower power consumption than CARD, quantifiable in a 17.7% decrease for the Gaussian traces and in a 11.8% decrease in the Multiple Gaussian traces where the change in periodicity leads to an additional challenge on the predictor.

Furthermore, it is possible to see that both CARD and ADTP consume less energy than PRWS. In particular, in the worst case of 20 km/h for both traces, ADTP consumes 89.6% and 91.6% less energy than PRWS, respectively for the Gaussian and Multiple Gaussian traces. Similarly, CARD consumes 95.2% and 95.4% less energy than PRWS in the Gaussian and Multiple Gaussian traces at 3.6 km/h. Concerning the wasted time energy product, both ADTP and CARD perform better than PRWS in the 3.6 km/h and 20 km/h cases. In the 40 km/h case, however, due to high randomness only CARD is capable to outperform both ADTP and PRWS with a 53.6% and 54.5% lower value for Gaussian and Multiple Gaussian traces, with PRWS achieving a performance edge on ADTP quantifiable in a 26.2% less for the Gaussian traces and a 7.5% less for the Multiple Gaussian traces. As mentioned, this is due to the high randomness in these scenarios, which poses great challenge to ADTP.

**Figure 12:**
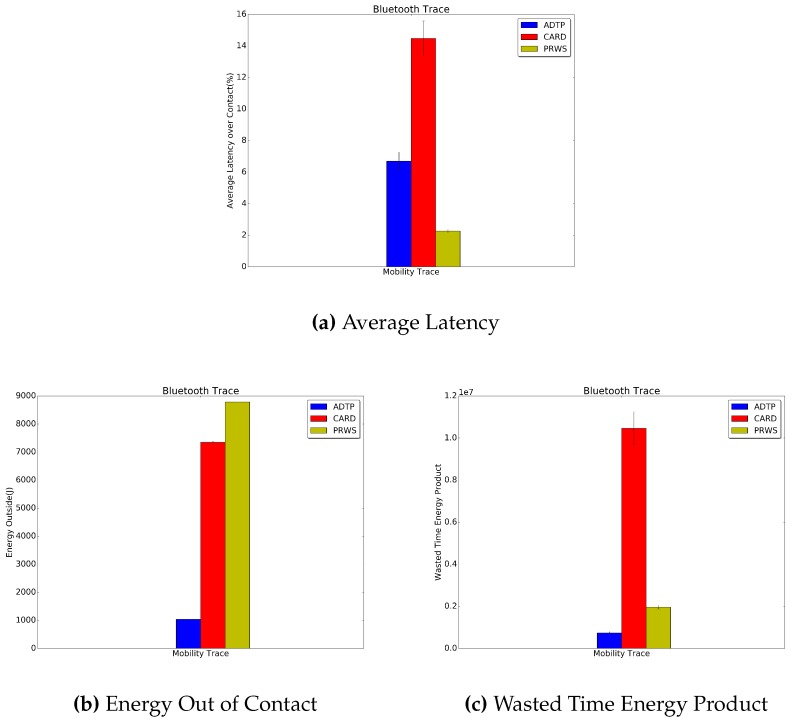
Bluetooth Traces Results.

**Figure 13:**
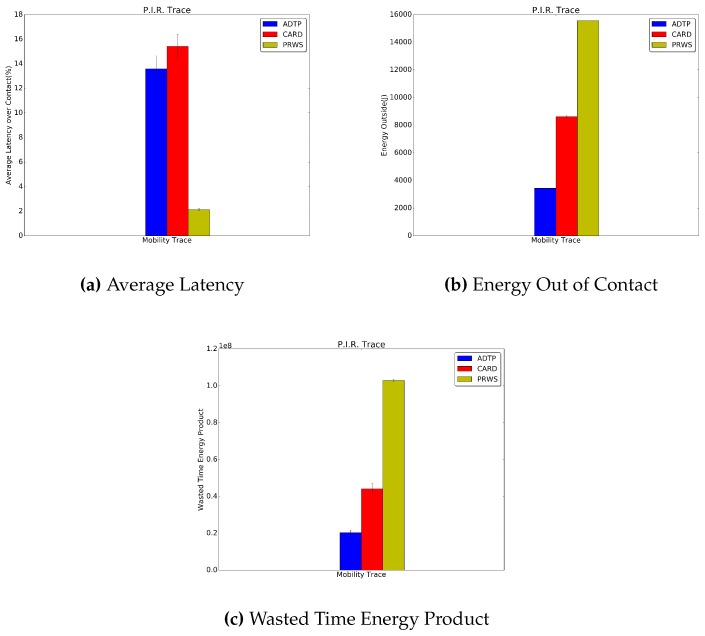
P.I.R. Traces Results.

Figure 12 and Figure 13 report the results for the traces collected during our in-house experiment. ADTP’s improvement over CARD in terms of average latency is of 7.8% and 1.8%, respectively for the Bluetooth and P.I.R. traces. Similarly for the energy consumption, ADTP saves 85.9% and 60.1% of power consumption with respect to CARD. Concerning the comparison with PRWS, in the Bluetooth traces, ADTP spends an energy with is 88.2% lower than PRWS, which combined with the wasted time leads to a 62.1% lower value for the wasted time energy product for ADTP. Similarly, in the P.I.R. traces, ADTP reaches a energy consumed which is 77.9% lower than PRWS. This value then translates into a combined wasted time energy product which allows ADTP to save 80.3% with respect to PRWS.

**Figure 14:**
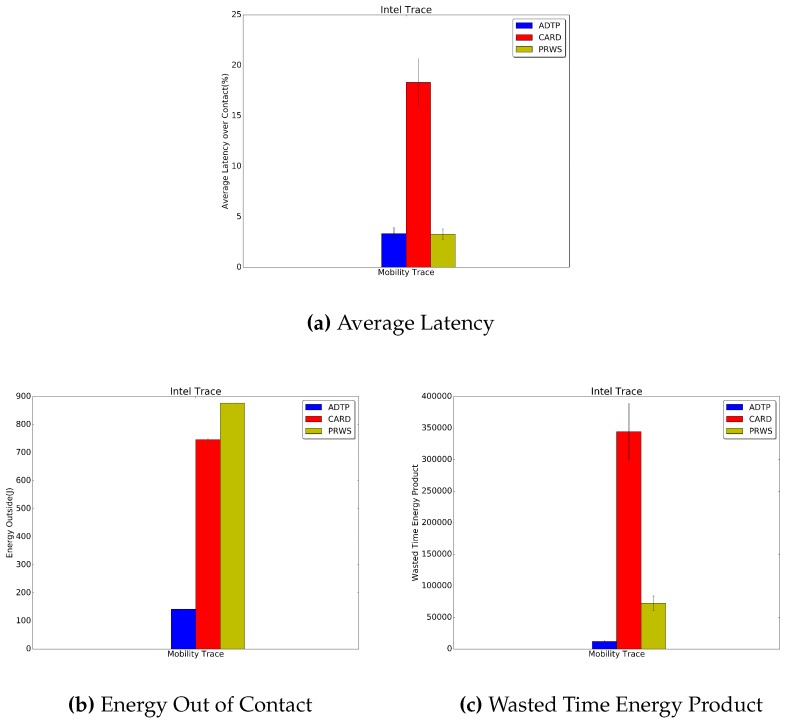
Intel Traces Results.

**Figure 15:**
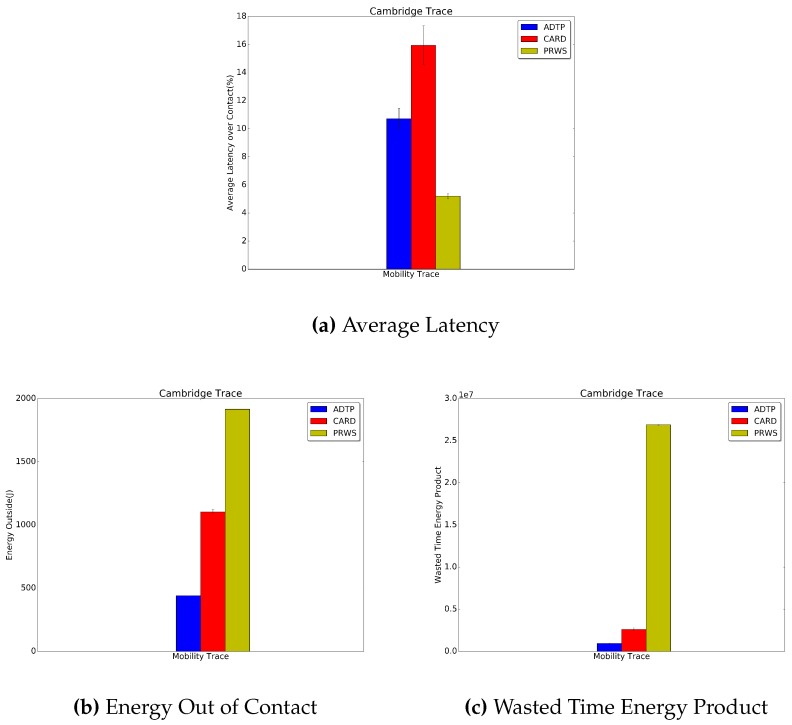
Cambridge Traces Results.

**Figure 16:**
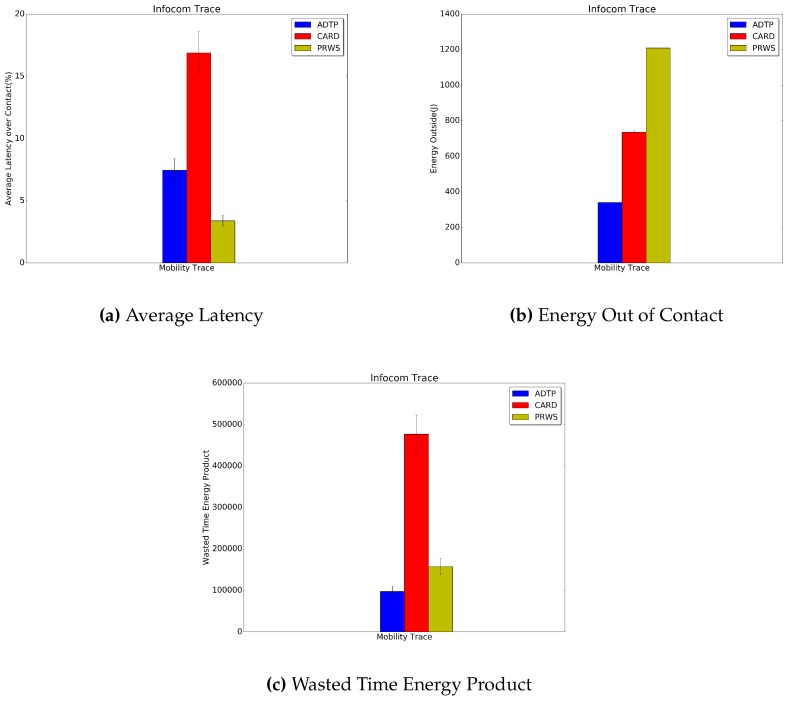
Infocom Traces Results.

Finally, in Figure 14, Figure 15 and Figure 16 the results for the Haggle project traces are presented. Concerning the latency for discovery, ADTP’s gain over CARD are quantifiable in 9.4% for Intel traces, 5.2% for Cambridge traces and 15% for Infocom traces.

Concerning the energy spent for discovery while not in contact, the performance edges of ADTP are of 53.7% for the Intel traces, 60.1% for the Cambridge traces and 81% for the Infocom traces. As regards the evaluation against PRWS, ADTP achieves a lower energy value as compared with PRWS, measured in 83.8% for the Intel traces, 77% for the Cambridge traces and 71.8% for the Infocom traces. Finally, the savings in wasted time energy product reached by ADTP is of 83.6% for the Intel traces, 96.5% for the Cambridge traces and 38% for the Infocom traces.

Across real world traces the best improvements of ADTP in terms of power consumption are for the Bluetooth traces (85% over CARD and 88.2% over PRWS) and this is due to the fine-grained resolution which allows to recognize also short contacts. However, the best latency advantage of ADTP over CARD is reported for the Infocom traces whereas the latency reduction shown by the Bluetooth traces roughly corresponds to the average across the real world traces, which is 7.8%.

This is most likely due to the difference in number of experienced contacts by the Bluetooth trace (≥600) with respect to the Infocom traces (≤50). Moreover, while PRWS achieves lower discovery latency in most cases, this is due to the fact that it stays awake for a considerably larger amount of time (well beyond the actual duration of the contact), which is illustrated by the amount of energy spent by PRWS which is the largest in most cases.

Finally, concerning the results about the percentage of discoveries across the total number of contacts experienced, we omit the representation since across all traces both CARD and ADTP show a very high percentage around 99% with a very little difference ±1%, which was similarly achieved in PRWS in all cases, except for the Cambridge traces in which the discovery ratio achieved is of 78.1%.

## 5. Conclusions

In this article, an arrival and departure time predictor for opportunistic IoT has been presented and evaluated. Results show that the proposed algorithm is capable to predict not only next but also future contact arrivals and duration with a good degree of accuracy. In addition, a discovery approach for scheduling communication based on such predictions has been introduced and evaluated against previous state-of-the-art protocols showing jointly improvements in terms of discovery latency and power consumption. In particular ADTP reaches a lower wasted time and energy for discovery if compared with previous state-of-the-art, as reported in the evaluation section, which as explained is indicative of a better overall performance. Moreover, ADTP adapts well to different mobility patterns, i.e., controlled periodic such as those experienced in robotised or micro aerial vehicle networks [53], periodic with variance such as those happening in urban public transportation scenarios and human mobility based.

Future plans for ADTP is to further enhance the predictor in order to not only optimize the scheduling for the next contact arrival, but also exploit multiple steps ahead prediction and knowledge of duration of contacts to further optimize not only the discovery but also the actual subsequent communication between devices. The knowledge of future contact durations could be exploited for example for tuning TCP networking or selecting the appropriate radio interface based on bandwidth requirements. Furthermore, prediction also allows optimization of message queues in IoT devices and duration-based caching for opportunistic content dissemination. Moreover, instead of adopting “greedy” schedulers, selection of most favourable (i.e., longer) contacts could also happen based on knowledge of multiple steps ahead mobility behaviour.

Knowledge about popularity, community membership and social relations as well as information about location tagging, which do not require additional energy or additional hardware to be generated, could also be exploited outside a data mining context and used to learn and predict *online* next arrival and departure times with a higher accuracy further improving our approach. Indeed, additional mobility features could be integrated in the function approximation model to build a more complex representation of the value function and the learning model kernelised to be able to express non-linear relations in the data, thus leading to predictions exploiting multiple sources of knowledge.

Furthermore, temporal knowledge about mobility, combined also with spatial information, could be provided “as-a-service” in the cloud to manage replication and migration of cloud hosted functionalities to match mobility patterns for opportunistic offloading of computation in opportunistic IoT following the edge computing paradigm.

Finally, as future work, we plan also to port our implementation of ADTP on dedicated real world hardware devices such as mobile and static IoT devices (i.e., motes or smartphones) in order to enable its exploitation by applications that can take advantage of Opportunistic IoT.

## Figures and Tables

**Table 1:** One step ahead accuracy of arrival and departure times prediction.

Traces	$t_{A_{k}}$ (%)					$t_{D_{k}}$ (%)			
	1 m	5 m	10 m	15 m		1 m	5 m	10 m	15 m
Deterministic	99.79	99.79	99.79	99.79		99.79	99.79	99.79	99.79
Multiple Deterministic	96.38	98.41	98.55	99.13		96.38	98.41	98.55	99.13
Gaussian	9.90	46.15	76.46	91.67		9.69	46.35	76.35	91.56
Multiple Gaussian	8.41	47.68	78.84	94.06		8.41	47.68	78.84	94.06
Bluetooth	30.73	82.48	90.92	93.47		29.30	82.01	91.88	93.95
P.I.R.	4.95	23.00	48.88	67.41		3.51	24.44	48.72	67.25
Intel	5.71	25.71	57.14	77.14		5.71	31.43	60.00	77.14
Cambridge	3.85	26.15	57.69	67.69		6.15	30.00	55.38	71.54
Infocom	11.11	31.11	44.44	51.11		11.11	37.78	46.67	55.56

**Table 2:** Two steps ahead accuracy of arrival and departure times prediction.

Traces	$t_{A_{k+1}}$ (%)					$t_{D_{k+1}}$ (%)			
	1 m	5 m	10 m	15 m		1 m	5 m	10 m	15 m
Deterministic	95.73	95.73	95.73	95.73		95.73	95.73	95.73	95.73
Multiple Deterministic	91.88	92.75	94.06	94.06		91.88	92.75	94.06	94.06
Gaussian	7.92	36.67	66.77	83.54		7.92	36.56	66.56	83.54
Multiple Gaussian	8.70	39.13	64.78	82.17		8.70	39.13	64.78	82.17
Bluetooth	15.45	67.04	81.69	86.46		16.72	66.72	82.17	87.58
P.I.R.	3.04	14.86	29.07	39.62		4.31	14.70	29.23	39.94
Intel	2.86	11.43	25.71	40.00		2.86	11.43	25.71	40.00
Cambridge	3.08	13.85	30.00	47.69		3.85	13.08	29.23	46.92
Infocom	4.44	20.00	33.33	40.00		4.44	24.44	37.78	42.22
